# P-2056. Modelling the Relative Vaccine Effectiveness of ARCT-154 versus BNT162b2 using Immunogenicity Data

**DOI:** 10.1093/ofid/ofae631.2212

**Published:** 2025-01-29

**Authors:** Van Nguyen, Pascal Crepey, Jean Marie Pivette, Ettan Settembre, Sankarasubramanian Rajaran, John Youhanna, Aimee Ferraro, Cheng Chang, Josephine Van Boxmeer, Joaquin F Mould-Quevedo

**Affiliations:** VHN Consulting, Montreal, Quebec, Canada; University of Rennes, Rennes, Bretagne, France; VHN Consulting, Montreal, Quebec, Canada; CSL Seqirus USA, Boston, Massachusetts; CSL Seqirus UK, Maidenhead, England, United Kingdom; CSL Seqirus USA, Boston, Massachusetts; CSL Seqirus USA, Boston, Massachusetts; CSL Seqirus USA, Boston, Massachusetts; CSL Seqirus USA, Boston, Massachusetts; CSL Seqirus Inc., Summit, New Jersey

## Abstract

**Background:**

Vaccination has become an essential weapon in the global battle against COVID-19. While showing high effectiveness against the original Wuhan-Hu-1 strain and early SARS-CoV-2 variants, questions have surfaced regarding the duration of immunity they provide, especially with the emergence of new viral strains. The innovative self-amplifying (SA)mRNA vaccine, ARCT-154, has demonstrated clinical improvements in extending duration of immune response. Our objective was to compare the long-term efficacy of ARCT-154 with BNT162b2 in an adult population by assessing relative vaccine effectiveness (rVE) over time.

**Methods:**

rVE was estimated using a two-step approach. First, we used geometric mean titer data (Days 29, 91, and 181 post-vaccination) from a phase 3 double-blind controlled clinical trial comparing the immune response of ARCT-154 and BNT162b2 against Wuhan-Hu-1 and Omicron BA.4/5 in healthy 18–77-year-olds (Japan Registry for Clinical Trials identifier: jRCT2071220080). We then incorporated this information into a published model correlating immunogenicity and efficacy to calculate the rVE of ARCT-154 versus BNT162b2 in symptomatic and severe cases.

**Results:**

ARCT-154 exhibited an increasing rVE against symptomatic infection with Wuhan-Hu-1 over time, ranging from 2.11% on Day 29 to 8.51% on Day 181 post-vaccination. rVE against symptomatic Omicron infection was 0% on Day 29 but increased to 13.85% by Day 91 and 20.25% by Day 181. rVE against severe illness ranging from 1% to 4% for Wuhan-Hu-1 and 1% to 13% for Omicron across all measured time points.

**Conclusion:**

ARCT-154 showed longer-lasting protection than BNT162b2 against symptomatic and severe COVID-19 for both the original Wuhan-Hu-1 and Omicron BA.4/BA.5 strains.

**Disclosures:**

Van Nguyen, PhD, CSL Seqirus USA: Advisor/Consultant Pascal Crepey, n/a, CSL Seqirus USA: Advisor/Consultant Jean Marie Pivette, n/a, CSL Seqirus USA: Advisor/Consultant Ettan Settembre, n/a, CSL Seqirus USA: Stocks/Bonds (Private Company) Sankarasubramanian Rajaran, n/a, CSL Seqirus UK: Stocks/Bonds (Private Company) John Youhanna, n/a, CSL Seqirus USA: Stocks/Bonds (Private Company) Aimee Ferraro, n/a, CSL Seqirus USA: Stocks/Bonds (Private Company) Cheng Chang, n/a, CSL Seqirus USA: Stocks/Bonds (Private Company) Josephine Van Boxmeer, n/a, CSL Seqirus Amsterdam: Stocks/Bonds (Private Company) Joaquin F. Mould-Quevedo, PhD, CSL Seqirus USA: Stocks/Bonds (Private Company)

